# Colobome cristallinien unilatéral: à propos d’un cas

**DOI:** 10.11604/pamj.2013.16.1.3150

**Published:** 2013-09-02

**Authors:** Hanan Handor, Rajae Daoudi

**Affiliations:** 1 Université Mohammed V Souissi, Service d’Ophtalmologie A de l’hôpital des spécialités, Centre hospitalier universitaire, Rabat, Maroc

**Keywords:** Colobome cristallinien, nystagmus, cataracte, Colobome of the eye lens, nystagmus, cataract

## Image en médicine

Nous rapportons le cas d’une jeune fille de 7 ans amenée par ses parents pour prise en charge d’une malvoyance qu’ils ont constaté depuis l’âge de 2 ans. L’examen à l’admission retrouve un nystagmus horizontal pendulaire. La réfraction automatique révèle une myopie de -7.5 dioptries en OD (l’œil droit) et de -6 dioptries en OG (l’œil gauche) avec une meilleure acuité corrigée à compte les doigts de près en ODG. L’examen du segment antérieur note un colobome irien bilatéral, associé au niveau de l’œil droit à un defect zonulaire inférieur allant de 5h à 7h avec une encoche cristallinienne en regard, et une cataracte polaire postérieure. L’examen du fond d’oeil a révélé un colobome papillaire et choriorétinien bilatéral très étendu. L’examen général avec réalisation d’une IRM cérébro-orbitaire sont revenus négatifs. Les colobomes sont des malformations congénitales de l’œil secondaires à une anomalie de fermeture de la fente fotale. Toutes les structures de l’oil peuvent être touchées. Toutefois Le colobome cristallinien reste une localisation extrêmement rare de cette malformation, notre cas s’ajoute aux rare cas décrits dans la littérature. Comme toutes les formes du colobome, il peut s’associer à des malformations oculaires comme la cataracte, la microphtalmie ou l’ectopie cristallinienne qui constitue d’ailleurs un des diagnostics différentiels du colobome cristalliniens. Des malformations extra oculaires peuvent aussi y être associées, notamment d’ordre neurologique (malformations du corps calleux) ce qui impose un bilan général clinique et paraclinique.

**Figure 1:**
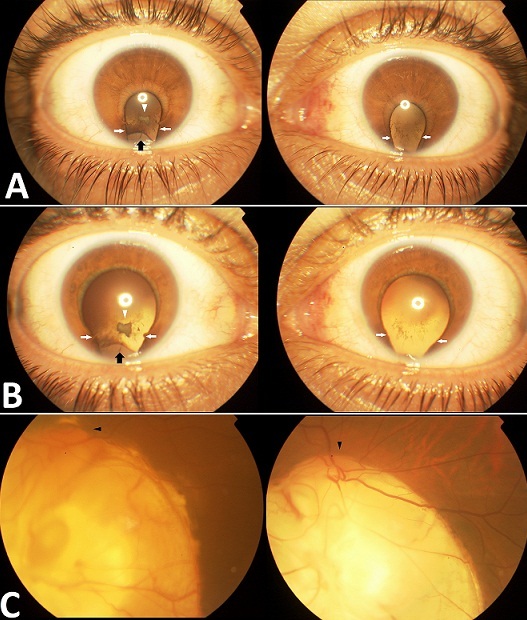
A) aspect avant la dilatation pupillaire montrant un colobome irien bilatéral (flèches blanches) associé à une cataracte polaire postérieure (tête de flèche blanche) et un colobome cristallinien de l’œil droit qui apparait sous forme d’une encoche à concavité inférieure du bord du cristallin (flèche noire) B) aspect après dilatation (notez le reflet jaunâtre de la lueur pupillaire au niveau des deux yeux); C) aspect du fond d’œil montrant un colobome papillaire et choriorétinien bilatéral étendu aux papilles optiques (tête de flèche noire).

